# Factors Associated with Timely First-Dose Pentavalent and Measles–Rubella Vaccination: A Cross-Sectional Study in East New Britain, Papua New Guinea

**DOI:** 10.3390/vaccines13020156

**Published:** 2025-02-04

**Authors:** Milena Dalton, William Pomat, Margie Danchin, Caroline S. E. Homer, Benjamin Sanderson, Patrick Kiromat, Leanne J. Robinson, Michelle J. L. Scoullar, Pele Melepia, Moses Laman, Hannah A. James, Elsie Stanley, Edward Waramin, Stefanie Vaccher

**Affiliations:** 1 Burnet Institute, 85 Commercial Rd, Melbourne, VIC 3004, Australia; 2 Faculty of Medicine, Dentistry and Health Sciences, University of Melbourne, 161 Barry St, Carlton, VIC 3010, Australia; 3 Papua New Guinea Institute of Medical Research, Goroka P.O. Box 60, Papua New Guinea; 4 Kirby Institute, Wallace Wurth Building, High St, University of New South Wales, Sydney, NSW 2052, Australia; 5 Vaccine Uptake Group, Murdoch Children’s Research Institute, Royal Children’s Hospital, 50 Flemington Road, Parkville, VIC 3052, Australia; 6 Department of General Medicine, The Royal Children’s Hospital, 50 Flemington Road, Parkville, VIC 3052, Australia; 7 East New Britain Provincial Health Authority, Butuwin, Kokopo P.O. Box 714, Papua New Guinea; 8 The School of Public Health and Preventive Medicine, Monash University, 553 St Kilda Road, Melbourne, VIC 3004, Australia; 9 Papua New Guinea Institute of Medical Research, Boroko, National Capital District 111, Kokopo P.O. Box 60, Papua New Guinea; 10 Burnet Institute, Section 90, Lot 1 Takubar, Kokopo P.O. Box 1458, Papua New Guinea; 11 Papua New Guinea National Department of Health, Aopi Building Centre, Waigani Dr, National Capital District 121, Port Moresby P.O. Box 807, Papua New Guinea

**Keywords:** zero dose, routine immunization, Papua New Guinea, health system strengthening, timely vaccination

## Abstract

Background: Immunization coverage varies across Papua New Guinea. In East New Britain (ENB) Province in 2022, only 65.5% and 50.2% of children under one year received their first dose of pentavalent (DTP1) and measles–rubella (MR1) vaccine, respectively. This study aimed to examine barriers and enablers to routine immunization in areas of un(der)-vaccination in ENB. Methods: A face-to-face survey was conducted with caregivers of children aged 12–23 months in ENB. We used Poisson regression to calculate incidence rate ratios (IRR) and 95% confidence intervals (95% CI) for factors associated with timely receipt of DTP1 or MR1 vaccines, defined as a child who was vaccinated between –2 and +30 days of the vaccine schedule. Delayed receipt is defined as a child who was vaccinated >30 days from the recommended due date. Results: Among 237 caregivers surveyed, 59.9% of children were vaccinated within the “timely” window for DTP1 and 34.1% for MR1. Timely DTP1 receipt was associated with a facility-based birth (IRR:1.93; 95% CI: 1.10–3.38) and trusting healthcare workers “very much”, compared to “a little or moderately” (IRR:1.53; 95% CI: 1.17–1.99). For MR1, the caregiver having completed tertiary/vocational education (IRR:1.79; 95% CI: 1.15–2.78), reporting taking a child to be vaccinated is affordable (IRR:1.52; 95% CI: 1.04–2.22), and healthcare workers explaining immunization services and answering associated questions (IRR:1.68; 95% CI: 1.18–2.41) were associated with timely vaccination. Conclusions: Activities to improve timely vaccination in ENB could include strengthening healthcare worker interpersonal communication skills to optimize trust and incentivizing women to give birth in a health facility.

## 1. Introduction

Routine immunization continues to be one of the most life-saving and cost-effective public health interventions and is essential for the promotion of child health [1,2]. Since it was established in 1974, the World Health Organization (WHO) Expanded Programme on Immunisation has averted an estimated 146 million deaths of children under five globally [3]. However, the benefits of immunization remain inequitable, and coverage rates vary widely among countries within the six WHO regions [4]. This inequity was further highlighted during the COVID-19 global pandemic [5].

Along with vaccine coverage, timely vaccine receipt is essential to prevent serious illness and death and maintain population immunity against vaccine-preventable diseases [6]. Timely vaccine receipt is defined as receiving a scheduled vaccine within 30 days of the recommended receipt date [7]. The national immunization schedule in each country specifies the age each vaccine dose should be administered [8]. Previously identified factors that can prevent timely vaccine receipt in low-resource settings include socioeconomic status, difficulty accessing health services, healthcare worker shortages, and challenges with the quality of service provision [9].

Papua New Guinea (PNG) introduced the Expanded Programme on Immunisation in 1981, and it now includes 11 vaccines (Table 1) [10]. It is implemented in each of the 21 provinces and the Autonomous Region of Bougainville through government and religious organization managed facilities and associated outreach programs [10]. Within the Western Pacific Region, PNG reports low childhood vaccination rates which have declined further with the COVID-19 pandemic [11,12]. In 2023, PNG’s coverage for first-dose pentavalent vaccine (DTP1) was 45%, and 44% for first-dose measles–rubella (MR1) vaccine [11]. In East New Britain Province in 2022, 34.5% of children were zero-dose children (children who did not receive DTP1) and only half (50.2%) of East New Britain’s children received MR1 [13,14]. Without strengthened routine immunization services, the children of East New Britain will continue to be at increased risk of illness or death from vaccine-preventable diseases. This study aimed to examine barriers and enablers to routine immunization in areas of un(der)-vaccination in East New Britain.

## 2. Materials and Methods

### 2.1. Study Design

We conducted a cross-sectional study in Gazelle and Kokopo districts of East New Britain Province, PNG, between February and September 2023 to examine barriers and enablers to routine immunization. This study had four components: (i) health facility survey; (ii) quantitative and qualitative surveys with caregivers of children aged 9 to 23 months; (iii) interviews with healthcare workers; and (iv) focus group discussions with community and religious leaders. This analysis reports on the quantitative survey data to explore factors associated with children aged 9 to 23 months receiving DTP1 and MR1 vaccines on time.

### 2.2. Study Setting

East New Britain is the most populous province in the Islands Region of PNG with 457,169 people, the majority of whom (approximately 301,538) live in the Kokopo and Gazelle districts where this study was conducted [15]. Five local-level government areas, one urban and four rural, were identified for study inclusion following mapping of un- and under-immunized children using health facility coverage data from 2015 to 2021 and consultation with the East New Britian Provincial Health Authority [16]. Within Gazelle District, we included Inland Baining Rural and Toma/Vunadidir Rural local-level government areas. Kokopo District included Bitapaka Rural, Duke of York Rural, and Kokopo/Vunamami Urban local-level government areas. Routine immunization services in East New Britain are delivered at health facilities, through one-day mobile outreach clinics and overnight outreach patrols at the community level [16]. In 2023, 28 outreach activities were undertaken per 1000 children under five years of age [13].

### 2.3. Participants

Adult caregivers of a child aged 9 to 23 months residing in the study areas were eligible to participate in this study. A sample size of 250 children (50 per local-level government area) was calculated using immunization coverage and census population data. Caregivers were randomly recruited from villages within each local-level government area. To do this, data collectors went to every second house. Eligible caregivers from each village were invited to participate in this study. Informed written consent was collected from each participant before any data were collected. If a caregiver had multiple children aged between 9 and 23 months, they only reported on their oldest child within that age range.

### 2.4. Data Collection

We developed a survey to collect social and behavioral insights to routine childhood immunization, informed by previous surveys used to capture social and behavioral insights to routine childhood immunization as well as the WHO Behavioural and Social Drivers of Vaccination tool [17,18,19,20]. The survey was translated into Tok Pisin by PNG translation company. The Tok Pisin translation was further adapted to the local context in consultation with healthcare workers, community members, and local researchers during the pilot testing phase.

Research officers received training on implementing the survey in two workshops. This training included a discussion to reflect on beliefs or expectations that may influence the interview to reduce interviewer bias. Research officers collected data from participants in a face-to-face interview using REDCap software v13.3.0 installed on electronic tablets and hosted on Burnet’s institutional server. The survey was available in both English and Tok Pisin and used branching logic and validation rules where appropriate to strengthen data collection quality. Vaccination status was checked by reviewing records in the child health book when available and asking the caregiver to recall when the child received their vaccines.

### 2.5. Operational Definitions

For the purposes of this analysis, the following definitions were used:

**Timely vaccination:** a child who is vaccinated from two days before the vaccine due date as per the PNG immunization schedule and within 30 days after the recommended due date (Table 1) [7].

**Delayed vaccination:** a child who was vaccinated later than 30 days from the recommended due date (Table 1) [7].

### 2.6. Outcome

This study analyzed two outcomes of interest. Outcome one is timely receipt of DTP1, which is defined as administered to a child between 28 and 60 days from birth. Outcome two is timely receipt of MR1, which is defined as administered to a child between 178 and 210 days from birth. Children were also classified as delayed if the child had received the vaccine, but no date was available via recall or from the date recorded in the child health book. Analyses were conducted using Stata v17.0 (StataCorp, College Station, TX, USA).

### 2.7. Co-Variates

We collected demographic data capturing participant location, education level, sex, and age. For education, we grouped responses into “primary or less”, “high/secondary”, and “tertiary/vocational”. Disability questions were adapted from the Washington Group on Disability Statistics questions, which asked about any difficulties with seeing, hearing, or movement [21]. We grouped caregiver and child living with a disability responses into “no disability” or “disability”.

We used a four-point Likert scale to measure trust in healthcare workers. We grouped “a little” and “moderately”. The number of participants in the “a little” category did not provide a meaningful comparison. Combining “a little” with “moderately” allowed for a meaningful comparison to be made. No participants selected “not at all” so this category was removed from the analysis.

A three-point Likert scale was to measure affordability of taking a child to be vaccinated. We grouped “not at all affordable” and “somewhat affordable” together as a comparison to “affordable”, which allowed for us to investigate any participants that noted any issues with affordability, noting that vaccines are administered free of charge in PNG. We grouped clinic wait time into “less than one hour”, “between 1 h and less than two hours”, and “greater than two hours”.

### 2.8. Data Analysis

Timely DTP1 and MR1 receipt was analyzed using an exponentiated Poisson regression model. We chose this generalized linear model (Poisson distribution) because of the distribution of the outcomes. Co-variates for the modelling were chosen from the previous literature and expert guidance of the variables of interest [9,22]. Sociodemographic characteristics were analyzed using descriptive statistics. The univariate and multivariate incidence rate ratio (IRR) and 95% confidence intervals (95% CI) are reported. Participants who had missing data on the outcome or any of the co-variates in the modelling were excluded.

## 3. Results

### 3.1. Descriptive Data

A total of 237 caregivers met the eligibility criteria and provided informed consent. Demographic characteristics are reported in Table 2. Almost half (49.4%; n = 117) of caregivers reported an education level of primary school or less. Most caregivers were mothers (87%; n = 206) and lived in rural areas (86.9%; n = 206). Most participants were aged between 20 and 30 years (62.9%; n = 149). Among caregivers, 54% reported household duties as their form of employment, followed by gardener/farmer at 24.1%. There were slightly more male children (53.2%; n = 126) reported. Twelve percent (29) of caregivers reported living with a disability, but very few children were reported as living with a disability (1.3%; n = 3).

Outcome data for DTP1 was available for 91% (215) participants and 75% (179) for MR1. Overall, 59.9% (142) of children were vaccinated within the “timely” window for DTP1 vaccine and 34.1% (81) for MR1 vaccine (Table 2). Of the 215 caregivers who provided outcome data for DTP1, 96% (206) identified as female and 77% (159) of these 206 females gave birth in a health facility. Of the 179 caregivers who provided outcome data for MR1, 72% (171) identified as female and 78% (133) of these 171 females gave birth in a health facility.

Of the 142 children who received DTP1 with a recorded date within the timely window, 16.2% (23) were ascertained through checking the child health book record, 78.9% (112) through checking the child health book record alongside parental recall, and 4.9% (7) through parental recall only. Of the 81 children who received MR1 with a recorded date inside the timely window, 12.3% (10) were ascertained through checking the child health book record, 80.2% (65) through checking the child health book record alongside parental recall, and 7.4% (6) through parental recall only.

### 3.2. Factors Associated with DTP1 Timeliness

One hundred and seventy-nine (75%) participants were included in the DTP1 timeliness analysis; those with missing data for the outcome or co-variates of interest were excluded. This analysis shows that timely receipt of DTP1 is significantly positively associated with a facility-based birth and having trust in healthcare workers (Table 3). A mother giving birth at a health facility was 93% more likely to have their child vaccinated on time for DTP1, compared to mothers who did not give birth at a health facility. Higher levels of trust in healthcare workers increases the likelihood of getting their child vaccinated with DTP1 by 53%, compared to caregivers who trusted healthcare workers a little or moderately.

### 3.3. Factors Associated with MR1 Timeliness

A separate timeliness analysis for MR1 was completed, including 62% (147) of survey participants; those with missing data for the outcome or co-variates of interest were excluded. This analysis shows that timely receipt of MR1 is significantly positively associated with a caregiver having a higher education level, trust in healthcare workers, and a healthcare worker explaining immunization services and answering caregiver questions well (Table 4). A caregiver with a tertiary/vocational education was 79% more likely to get their child vaccinated on time for MR1 compared to caregivers who had primary-level education or less. Caregivers who reported that it is affordable to take a child to be vaccinated are 52% more likely to get their child vaccinated on time for MR1 compared to caregivers who said taking your child to be vaccinated is not at all affordable or somewhat affordable. A healthcare worker explaining immunization services and answering questions well increases the likelihood of getting their child vaccinated for MR1 by 68% compared to the reference category.

## 4. Discussion

This study describes factors associated with timely receipt of DTP1 and MR1 vaccine in (un)der-vaccinated areas of East New Britain Province. We found that 59.9% of children were vaccinated within the “timely” window for DTP1 vaccine and 34.1% for MR1 vaccine. Timely receipt of DTP1 vaccine was positively associated with a facility-based birth and trusting healthcare workers “very much” as compared to “a little or moderately”. Factors positively associated with timely receipt of MR1 vaccine were the caregiver having completed tertiary/vocational education, the caregiver reporting that it is affordable to take a child to be vaccinated, and healthcare workers explaining immunization services and answering associated questions.

Results show that a facility-based birth was positively associated with children receiving timely DTP1 vaccine. Only 42% of women in PNG gave birth at a health facility in 2023 [23]. This is slightly higher in East New Britain, with 52% of women who gave birth at a health facility in 2023 [23]. This finding is supported by two DTP1 and measles-containing vaccine timeliness studies conducted in Ethiopia, which similarly found that a facility-based birth increased the odds of vaccinating their child on time [24,25]. However, a study conducted in Pakistan found that compared with home births, those in a private or public health facility had a significantly lower odds ratio for timely vaccinations [26]. Our study along with the two studies conducted in Ethiopia show that a facility-based birth is likely to result in more opportunities to promote the importance of vaccinating their child in a timely manner [24,25].

We identified that caregivers trusting healthcare workers more was positively associated with children receiving timely DTP1, and this appears to be consistent with other studies across many settings. For example, a study in urban and rural areas of Kenya found that more trust in healthcare workers was associated with higher odds of vaccine completion [27]. A study conducted in the United States (US) that explored trust as a factor in vaccination uptake for pregnant women found that provider vaccine safety, medical knowledge and experience, along with beneficent intentions increase trust between the consumer and provider [28]. This study also emphasized the importance of providers establishing trust through empathy when discussing vaccines [28]. If caregivers trust healthcare workers, they may be more likely to access healthcare services [28]. More studies looking at vaccination timeliness in low-middle income countries (LMICs) should consider including trust in healthcare workers as an area of interest.

Education has also been found in some settings to be positively associated with a higher likelihood of timely vaccination in children. A study conducted in Ethiopia found that a mother/caregiver who attended primary or secondary was six times more likely to vaccinate their child on time when looking across all scheduled routine vaccines [24]. For education past secondary school level, the mother/caregiver was five times more likely to vaccinate their child [24]. Another study conducted in Ethiopia that explored factors associated with vaccination status for children aged 12–23 months found that a mother who attained a secondary or above education was three times more likely to get their child vaccinated in a rural area and 2.8 times more likely in an urban area [29]. However, another study conducted in Ethiopia found that education status was not a predictor of timely MR1 [30]. Our study along with the two of the studies conducted in Ethiopia support the notion that higher levels of education also increase the likelihood of timely vaccination.

Our study found that affordability of taking a child to be vaccinated is positively associated with a child receiving MR1 on time. Studies conducted in LMICs and high-income countries have found financial accessibility barriers impact vaccination uptake. A study conducted in Burkina Faso found socioeconomic status impacts timely vaccination [31]. Similarly, a study conducted in South Africa found that healthcare professionals and caregivers consider financial constraints to vaccinating their child(ren) as an access barrier [32]. This finding is supported by a study conducted in Kenya, which found that financial constraints to receiving healthcare, including immunization, included the inability to pay for transportation to the facility [31,33]. A systematic review of socioeconomic differences in childhood vaccination in high-income countries found that travel and time costs of taking the child to be vaccinated is an access barrier [34]. It is important to consider the affordability of a taking a child to be vaccinated, such as transport costs, taking time off work, or finding child care, when examining barriers to timely vaccination.

In our study, healthcare workers explaining immunization services and answering associated questions well was positively associated with children receiving timely MR1 vaccine. The Kenyan study that examined what caregiver-level factors are associated with children’s vaccination status found that people who reported higher patient-centered quality of vaccination care had lower odds of having children with delayed or missed vaccinations [27]. A study conducted in the US found that the biggest proportion of parents who changed their minds about delaying or not getting a vaccination for their child listed information or assurances from healthcare provider as their main reason [35]. Findings from another study conducted in the US highlighted the importance of a doctor’s opinion surpassing concerns about vaccines [28]. However, a study conducted in Tanzania found no difference in vaccine uptake based on a mother’s perception of the vaccine provider–client relationship [36]. Healthcare workers explaining immunization services and answering associated questions can mean that the caregiver is more informed, and trust is built. More studies looking at vaccination timeliness in LMICs should consider collecting data on healthcare workers explaining immunization services and answering associated questions.

There are some limitations to this study. Sites were identified through mapping of zero-dose and under-immunized children across East New Britain using health facility DTP1, DTP3, and MR1 coverage data, alongside extensive consultation with the East New Britian Provincial Health Authority and healthcare workers to guide feasibility and site selection. Feasibility considerations included law and order challenges; study staff safety and working conditions, including modes of transport; and funding needed to reach remote areas. These feasibility considerations mean that our sample may be biased towards more accessible locations and caregivers who were more accessible and engaged with the healthcare system. This may mean that the proportion of children who were immunized is over-represented. Increased funding to reach remote areas or to return to the same site at different periods could be considered to address these limitations in future studies. Participant answers may be influenced by social desirability bias, with participants potentially reporting more socially desirable health seeking behaviors during interviews. Additionally, we are only able to present the association of co-variates to the two outcomes of interest due to the small sample size.

This analysis is part of the first cross-sectional survey implemented to capture barriers and enablers to routine childhood immunization in the five local-level government areas in Gazelle and Kokopo Districts in East New Britain, PNG. Data were collected from urban, rural, inland, and small island communities to enable some generalizability to other similar settings. Therefore, although these results are specific to the East New Britain context, it is likely that similar factors underlie timeliness of vaccination in other areas of PNG, and this should be further explored.

## 5. Conclusions

This study found that timely receipt of DTP1 vaccine was positively associated with a facility-based birth and trusting healthcare workers “very much”. For MR1 vaccine, we found that factors positively associated with timely receipt were the caregiver having higher levels of education, affordability of taking a child to be vaccinated, and good healthcare worker communication. A health system-strengthening approach is needed to improve routine immunization delivery and timely receipt in support of the PNG National Department of Health 2021–2025 Maternal and Newborn Health strategy. Activities could include strengthening healthcare worker interpersonal skills to build trust between caregivers and healthcare workers, and incentivizing women to give birth in a health facility. These activities should be considered as part of the routine immunization programming in the province, with targeted implementation guided by identified needs and reaching priority un(der)-vaccinated populations.

## Figures and Tables

**Table 1:** Vaccination Schedule in PNG.

Vaccine	Age
BCG *(vaccine to prevent tuberculosis infection)*	Birth
HepB *(vaccine to prevent hepatitis B infection)*	Birth
DTP Hib HepB *(vaccine to prevent diphtheria, tetanus, pertussis, Haemophilus influenzae type B, and hepatitis B infections)* ^1^	1, 2, 3 months
IPV *(inactivated poliovirus given by injection to prevent polio infection)*	3, 9 months
MR *(vaccine to prevent measles and rubella infection)*	6, 9, 18 months
OPV *(weakened poliovirus given by mouth to prevent polio infection)*	1, 2, 3 months
Pneumococcal *(vaccine to prevent Streptococcus pneumoniae infection)*	1, 2, 3 months
TT *(Tetanus toxoid vaccination to prevent tetanus infection)*	7, 13 years and in pregnancy

^1^ Pentavalent vaccine.

**Table 2:** Descriptive statistics (N = 237).

	Category	Frequency (%)
DTP1		
	Timely	142 (59.9)
	Delayed	73 (30.8)
	Missing	22 (9.3)
MR1		
	Timely	81 (34.2)
	Delayed	98 (41.4)
	Missing	58 (24.4)
Caregiver education level		
	Primary or less	117 (49.4)
	High/secondary	79 (33.3)
	Tertiary/vocational	40 (16.9)
	Missing	1 (0.4)
Urban/rural		
	Urban	31 (13.1)
	Rural	206 (86.9)
Caregiver relationship to the child		
	Mother	206 (87)
	Father	6 (2.5)
	Grandmother	14 (5.9)
	Aunty	9 (3.8)
	Older sibling Other family member	1 (0.4) 1 (0.4)
Caregiver age		
	18–20 years	4 (1.7)
	20–30 years	149 (62.8)
	31–40 years	64 (27.0)
	41–50 years	17 (7.2)
	50+ years	3 (1.3)
Caregiver employment		
	Gardener/farmer	57 (24.1)
	Small business owner	9 (3.8)
	Household duties	128 (54.0)
	Student	11 (4.6)
	Formal full-time employment	5 (2.1)
	Formal part-time employment	8 (3.4)
	Unemployed	17 (7.2)
	Other	2 (0.8)

**Table 3:** Factors associated with timely DTP1 receipt (n = 179).

	N	Univariate IRR	95% CI	Multivariate IRR	95% CI
Caregiver education level					
Primary or less	85	1 (reference)	-	1 (reference)	-
High/secondary	63	1.21	0.95, 1.53	1.11	0.88, 1.40
Tertiary/vocational	31	1.09	0.79, 1.50	0.95	0.68, 1.31
Urban/rural					
Urban	18	1 (reference)	-	1 (reference)	-
Rural	161	1.05	0.71, 1.55	1.07	0.74, 1.56
Child sex					
Male	95	1 (reference)	-	1 (reference)	-
Female	84	1.23	0.99, 1.53	1.23	0.98, 1.53
Mother gave birth at a health facility					
No	27	1 (reference)	-	1 (reference)	-
Yes	152	**2.09**	**1.21**, **3.60**	**1.93**	**1.10**, **3.38**
Mother had Td vaccine					
No	20	1 (reference)	-	1 (reference)	-
Yes	159	1.48	0.90, 2.43	1.16	0.69, 1.94
Affordability of taking your child to be vaccinated					
Not at all affordable or somewhat affordable	79	1 (reference)	-	1 (reference)	-
Affordable	100	1.14	0.91, 1.43	1.11	0.89, 1.38
Travel > 30 min to clinic					
No	41	1 (reference)	-	1 (reference)	-
Yes	138	1.01	0.78, 1.32	1.05	0.82, 1.35
Clinic wait time					
<1 h	63	0.95	0.69, 1.31	0.85	0.63, 1.15
1–<2 h	77	1.13	0.85, 1.52	1.11	0.84, 1.47
≥2 h	39	1 (reference)	-	1 (reference)	-
Vaccinated through outreach or mobile clinic					
No	68	1 (reference)	-	1 (reference)	-
Yes	111	0.95	0.76, 1.19	0.94	0.76, 1.17
Immunization service satisfaction					
No	60	1 (reference)	-	1 (reference)	-
Yes	119	1.06	0.83, 1.34	0.86	0.66, 1.11
Trust healthcare workers					
A little or moderately	73	1 (reference)	-	1 (reference)	-
Very much	106	**1.39**	**1.08**, **1.79**	**1.53**	**1.17**, **1.99**
Healthcare worker explains immunization services and answers caregiver questions well					
No	93	1 (reference)	-	1 (reference)	-
Yes	86	1.02	0.82, 1.27	0.98	0.78, 1.22

**Table 4:** Factors associated with timely MR1 receipt (n = 147).

	N	Univariate IRR	95% CI	Multivariate IRR	95% CI
Caregiver education level					
Primary or less	70	1 (reference)	-	1 (reference)	-
High/secondary	50	1.4	0.91, 2.13	1.33	0.88, 2.01
Tertiary/vocational	27	**1.65**	**1.06, 2.58**	**1.79**	**1.15, 2.78**
Urban/rural					
Urban	15	1 (reference)	-	1 (reference)	-
Rural	132	1.13	0.59, 2.17	1.47	0.76, 2.85
Child sex					
Male	77	1 (reference)	-	1 (reference)	-
Female	70	1.40	0.97, 2.01	1.42	1.00, 2.02
Mother gave birth at a health facility					
No	21	1 (reference)	-	1 (reference)	-
Yes	126	1.66	0.82, 3.36	1.10	0.54, 2.23
Mother had Td vaccine					
No	14	1 (reference)	-	1 (reference)	-
Yes	133	2.21	0.79, 6.14	1.67	0.58, 4.75
Affordability of taking your child to be vaccinated					
Not at all affordable or somewhat affordable	67	1 (reference)	-	1 (reference)	-
Affordable	80	**1.56**	**1.06, 2.31**	**1.52**	**1.04** **, 2.22**
Travel >30 min to clinic					
No	32	1 (reference)	-	1 (reference)	-
Yes	115	1.13	0.71, 1.80	1.34	0.87, 2.05
Clinic wait time					
<1 h	44	1.07	0.68, 1.69	0.94	0.62, 1.42
1–<2 h	70	0.79	0.50, 1.26	0.73	0.48, 1.09
≥2 h	33	1 (reference)	-	1 (reference)	-
Vaccinated through outreach or mobile clinic					
No	51	1 (reference)	-	1 (reference)	-
Yes	96	0.76	0.53, 1.09	0.82	0.58, 1.15
Immunization service satisfaction					
No	54	1 (reference)	-	1 (reference)	-
Yes	93	1.24	0.83, 1.84	0.82	0.53, 1.24
Trust healthcare workers					
A little or moderately	61	1 (reference)	-	1 (reference)	-
Very much	86	1.41	0.95, 2.10	1.46	0.98, 2.16
Healthcare worker explains immunization services and answers caregiver questions well					
No	81	1 (reference)	-	1 (reference)	-
Yes	66	**1.56**	**1.08**, **2.25**	**1.68**	**1.18, 2.41**

## Data Availability

The data presented in this study are available upon request from the corresponding author. Due to ethical restrictions, they are not publicly available.

## Abbreviations

The following abbreviations are used in this manuscript:

WHO	World Health Organization
PNG	Papua New Guinea
DTP	Pentavalent
MR	Measles-Rubella
IRR	Incidence Rate Ratio
CI	Confidence Interval

## References

[1] World Health Organization (2019). Immunization. https://www.who.int/news-room/facts-in-pictures/detail/immunization.

[2] World Health Organization (2022). Vaccines and Immunization. https://www.who.int/health-topics/vaccines-and-immunization#tab=tab_1.

[3] Shattock A.J., Johnson H.C., Sim S.Y., Carter A., Lambach P., Hutubessy R.C.W., Thompson K.M., Badizadegan K., Lambert B., Ferrari M.J. (2024). Contribution of vaccination to improved survival and health: Modelling 50 years of the Expanded Programme on Immunization. Lancet.

[4] World Health Organization (2020). Immunization Agenda 2030: A Global Strategy to Leave No One Behind.

[5] World Health Organization (2022). Immunization Coverage. https://www.who.int/news-room/fact-sheets/detail/immunization-coverage.

[6] World Health Organization Essential Programme on Immunization: Catch-Up Vaccination. https://www.who.int/teams/immunization-vaccines-and-biologicals/essential-programme-on-immunization/implementation/catch-up-vaccination.

[7] National Center for Immunisation Research and Surveillance Australia (2022). Annual Immunisation Coverage Report 2021.

[8] World Health Organization Immunization, Vaccines and Biologicals: WHO recommendations for routine immunization—Summary tables. https://www.who.int/teams/immunization-vaccines-and-biologicals/policies/who-recommendations-for-routine-immunization---summary-tables.

[9] Wariri O., Okomo U., Kwarshak Y.K., Utazi C.E., Murray K., Grundy C., Kampmann B. (2022). Timeliness of routine childhood vaccination in 103 low-and middle-income countries, 1978–2021: A scoping review to map measurement and methodological gaps. PLoS Glob. Public Health.

[10] Gowin E., Kuzma J., Januszkiewicz-Lewandowska D. (2021). Knowledge among the rural parents about the vaccinations and vaccination coverage of children in the first year of life in Papua New Guinea—Analysis of data provided by Christian health services. BMC Infect. Dis..

[11] World Health Organization (2023). Immunization Dashboard Papua New Guinea. https://immunizationdata.who.int/dashboard/regions/western-pacific-region/PNG.

[12] Morgan C.J., Saweri O.P.M., Larme N., Peach E., Melepia P., Au L., Scoullar M.J.L., Reza M.S., Vallely L.M., McPake B.I. (2020). Strengthening routine immunization in Papua New Guinea: A cross-sectional provincial assessment of front-line services. BMC Public Health.

[13] Papua New Guinea National Department of Health (2023). 2022 Sector Performance Annual Review.

[14] Papua New Guinea National Department of Health (2022). East New Britain Immunisation Coverage Data.

[15] Papua New Guinea National Department of Health (2023). National Population Estimate 2021.

[16] Burnet Institute (2023). Reaching Zero-Dose and Under-Immunised Children in East New Britain, Papua New Guinea: Year One Formative Data Collection Summary Results.

[17] Sinuraya R.K., Kusuma A.S.W., Pardoel Z.E., Postma M.J., Suwantika A.A. (2022). Parents’ Knowledge, Attitude, and Practice on Childhood Vaccination During the COVID-19 Pandemic in Indonesia. Patient Prefer. Adherence.

[18] Ali A.H.M., Abdullah M.A., Saad F.M., Mohamed H.A.A. (2020). Immunisation of children under 5 years: Mothers’ knowledge, attitude and practice in Alseir locality, Northern State, Sudan. Sudan. J. Paediatr..

[19] UNICEF, L. Sakvarelidze National Center for Disease Control and Public Health (2017). Immunization KAP Survey in the Country of Georgia, 2016 Final Report.

[20] World Health Organization (2022). Behavioural and Social Drivers of Vaccination: Tools and Practical Guidance for Achieving High Uptake.

[21] Washington Group on Disability Statistics (2022). Washington Group Short Set on Functioning.

[22] Menezes A.M.B., Flores T.R., Pereira A.M., Berrutti B., Marques G., Luquez K.Y.S., Brum L.W., Echeverry L.F.A., Freire M.B.O., Weisshahn N.K. (2022). Delay in quadrivalent vaccine (DTP+Hib) in children 12 to 23 months of age: Brazilian National Health Survey, 2013. Cad. Saúde Pública.

[23] Papua New Guinea National Department of Health (2024). 2023 Sector Performance Annual Review.

[24] Dejene H., Girma D., Geleta L.A., Legesse E. (2022). Vaccination timeliness and associated factors among children aged 12–23 months in Debre Libanos district of North Shewa Zone, Oromia Regional State, Ethiopia. Front. Pediatr..

[25] Dirirsa K., Makuria M., Mulu E., Deriba B.S. (2022). Assessment of vaccination timeliness and associated factors among children in Toke Kutaye district, central Ethiopia: A Mixed study. PLoS ONE.

[26] Noh J.-W., Kim Y.-m., Akram N., Yoo K.B., Cheon J., Lee L.J., Kwon Y.D., Stekelenburg J. (2019). Determinants of timeliness in early childhood vaccination among mothers with vaccination cards in Sindh province, Pakistan: A secondary analysis of cross-sectional survey data. BMJ Open.

[27] Moucheraud C., Ochieng E., Ogutu V., Sudhinaraset M., Szilagyi P.G., Hoffman R.M., Glenn B., Golub G., Njomo D. (2024). Trust in health workers and patient-centeredness of care were strongest factors associated with vaccination for Kenyan children born between 2017–2022. Vaccine X.

[28] Limaye R.J., Malik F., Frew P.M., Randall L.A., Ellingson M.K., O’Leary S.T., Bednarczyk R.A., Oloko O., Salmon D.A., Omer S.B. (2020). Patient Decision Making Related to Maternal and Childhood Vaccines: Exploring the Role of Trust in Providers Through a Relational Theory of Power Approach. Health Educ. Behav..

[29] Mastewal Worku L., Lucy B., Peter W., Mulugeta M., Ayalneh F. (2016). Factors for Low Routine Immunization Performance; A Community Based Cross Sectional Study in Dessie Town, South Wollo Zone, Ethiopia, 2014. Adv. Appl. Sci..

[30] Marefiaw T.A., Yenesew M.A., Mihirete K.M. (2019). Age-appropriate vaccination coverage and its associated factors for pentavalent 1-3 and measles vaccine doses, in northeast Ethiopia: A community-based cross-sectional study. PLoS ONE.

[31] Koulidiati J.L., Kaboré R., Nebié E.I., Sidibé A., Lohmann J., Brenner S., Badolo H., Hamadou S., Ouédraogo N., De Allegri M. (2022). Timely completion of childhood vaccination and its predictors in Burkina Faso. Vaccine.

[32] Burnett J.M., Myende N., Africa A., Kamupira M., Sharkey A., Simon-Meyer J., Bamford L., Guo S., Padarath A. (2024). Barriers to Childhood Immunisation and Local Strategies in Four Districts in South Africa: A Qualitative Study. Vaccines.

[33] Moucheraud C., Mboya J., Njomo D., Golub G., Gant M., Sudhinaraset M. (2022). Trust, Care Avoidance, and Care Experiences among Kenyan Women Who Delivered during the COVID-19 Pandemic. Health Syst. Reform.

[34] Bocquier A., Ward J., Raude J., Peretti-Watel P., Verger P. (2017). Socioeconomic differences in childhood vaccination in developed countries: A systematic review of quantitative studies. Expert Rev. Vaccines.

[35] Gust D.A., Darling N., Kennedy A., Schwartz B. (2008). Parents with Doubts About Vaccines: Which Vaccines and Reasons Why. Pediatrics.

[36] Chambongo P.E., Nguku P., Wasswa P., Semali I. (2016). Community vaccine perceptions and its role on vaccination uptake among children aged 12–23 months in the Ileje District, Tanzania: A cross section study. Pan Afr. Med. J..

